# Cancer-associated fibroblasts refine the classifications of gastric cancer with distinct prognosis and tumor microenvironment characteristics

**DOI:** 10.3389/fonc.2023.1158863

**Published:** 2023-06-19

**Authors:** Lei Gu, Dan Ding, Cuicui Wei, Donglei Zhou

**Affiliations:** ^1^ Department of General Surgery, Shanghai Tenth People’s Hospital, School of Medicine, Tongji University, Shanghai, China; ^2^ Department of Gastroenterology, Changhai Hospital, Navy/Second Military Medical University, Shanghai, China; ^3^ Affiliated Hospital of Youjiang Medical University for Nationalities, Baise, China; ^4^ Department of Gastric Surgery, Fudan University Shanghai Cancer Center, Shanghai, China; ^5^ Department of Oncology, Shanghai Medical College, Fudan University, Shanghai, China

**Keywords:** gastric cancer, cancer-associated fibroblasts, prognosis, weighted gene co-expression network analysis, machine-learning algorithms

## Abstract

**Background:**

Cancer-associated fibroblasts (CAFs) are essential tumoral components of gastric cancer (GC), contributing to the development, therapeutic resistance and immune-suppressive tumor microenvironment (TME) of GC. This study aimed to explore the factors related to matrix CAFs and establish a CAF model to evaluate the prognosis and therapeutic effect of GC.

**Methods:**

Sample information from the multiply public databases were retrieved. Weighted gene co-expression network analysis was used to identify CAF-related genes. EPIC algorithm was used to construct and verify the model. Machine-learning methods characterized CAF risk. Gene set enrichment analysis was employed to elucidate the underlying mechanism of CAF in the development of GC.

**Results:**

A three-gene (*GLT8D2, SPARC* and *VCAN*) prognostic CAF model was established, and patients were markedly divided according to the riskscore of CAF model. The high-risk CAF clusters had significantly worse prognoses and less significant responses to immunotherapy than the low-risk group. Additionally, the CAF risk score was positively associated with CAF infiltration in GC. Moreover, the expression of the three model biomarkers were significantly associated with the CAF infiltration. GSEA revealed significant enrichment of cell adhesion molecules, extracellular matrix receptors and focal adhesions in patients at a high risk of CAF.

**Conclusion:**

The CAF signature refines the classifications of GC with distinct prognosis and clinicopathological indicators. The three-gene model could effectively aid in determining the prognosis, drug resistance and immunotherapy efficacy of GC. Thus, this model has promising clinical significance for guiding precise GC anti-CAF therapy combined with immunotherapy.

## Introduction

Gastric cancer (GC) is the fifth most common cancer worldwide, accounting for approximately 5.7% of all new cancer cases in 2020. The highest incidence rates are found in Eastern Asia (China, Japan, and Korea), followed by Eastern Europe and South America (1–3). According to the World Health Organization (WHO), there were an estimated 1.03 million new cases and 783,000 deaths from gastric cancer in 2020. Several risk factors have been associated with an increased risk of developing gastric cancer, including chronic Helicobacter pylori infection, tobacco smoking, alcohol consumption, high-salt and low-fruit and vegetable diets, and a family history of gastric cancer (3). Regardless of the effect of gastroscopy screening and treatment to improve the condition, progression and metastasis mainly lead to the mortality in patients with GC. Gastric cancer often goes undetected until it has reached an advanced stage, which makes it difficult to treat. Symptoms such as abdominal pain, nausea, and weight loss are often nonspecific and can be attributed to other conditions, leading to delays in diagnosis. Besides, gastric cancer is a complex disease with a range of histological subtypes and molecular profiles, which makes treatment selection challenging. Additionally, the tumor microenvironment can be hostile, with a range of immune suppressive mechanisms that limit the efficacy of some treatments (4, 5). Treatment options for gastric cancer are limited, particularly for advanced disease. Surgery is the mainstay of treatment for early-stage disease, but in advanced cases, systemic therapy with chemotherapy, targeted agents, or immunotherapy may be the only option. Tumor cells in GC tissues and their immune and stromal environments constitute the tumor microenvironment (TME). Overall, the challenge of treating and managing gastric cancer lies in its complexity, late diagnosis, limited treatment options, high relapse rate, and the presence of co-morbidities. Increasing evidence reveals that tumor growth, metastasis, immunosuppression and drug resistance are significantly related to tumor matrix components in the TME (6–8).

The tumor microenvironment (TME) has emerged as a critical factor in the diagnosis and clinical management of GC (9). There has been significant progress in understanding the complex interactions between tumor cells and the surrounding microenvironment, including immune cells, stromal cells, and extracellular matrix components. Recently, in terms of diagnosis, several studies have shown that analysis of TME components such as tumor-infiltrating lymphocytes (TILs), stromal cells, and extracellular matrix components can provide prognostic information in patients with gastric cancer. For example, high levels of TILs have been associated with improved survival outcomes in some studies, while high levels of stromal cells and extracellular matrix components have been associated with poor prognosis (10). In terms of treatment, several approaches targeting the TME have been investigated in gastric cancer, including immune checkpoint inhibitors, stromal-targeting agents, and combination therapies. Immune checkpoint inhibitors such as pembrolizumab and nivolumab have shown promising results in clinical trials, particularly in patients with high levels of programmed death-ligand 1 (PD-L1) expression or microsatellite instability-high (MSI-H) tumors. Stromal-targeting agents such as pegvorhyaluronidase alfa (PEGPH20) and trabectedin have also shown efficacy in preclinical studies and early-phase clinical trials (11). Recent studies have shown that targeting CAFs using small molecule inhibitors or gene therapy can inhibit tumor growth in gastric cancer (10–12). Additionally, targeting stromal cells could improve the efficacy of chemotherapy and immunotherapy in GC (12). Thus, the TME has become an important area of research in GC, with significant progress made in understanding its role in diagnosis and treatment. Further research is needed to identify optimal biomarkers and treatment strategies that target the TME in gastric cancer.

As a key component of the tumor matrix, cancer-associated fibroblasts (CAFs) secrete several growth factors and cytokines, which are important for promoting tumor progression and migration (13, 14), stimulating epithelial-mesenchymal transition (EMT) (15, 16) and inducing chemical resistance (17, 18) and immunosuppression (19, 20). Additionally, the extracellular matrix (ECM) promotes tumor cell migration ability and inhibit anti-tumor leukocyte invasion. Moreover, CAFs can deposit and recombine ECM, leading to tumor progression, immune evasion and therapeutic resistance (21–24). Therefore, combining targeted CAF-mediated immunosuppressive matrix microenvironments with immunotherapy has the potential to improve the efficacy of immune checkpoint inhibitors (25). For example, fibroblast activating protein^+^ (FAP^+^) CAF depletion resulted in elevated CD8^+^ T cell infiltration and lower macrophage concentration, thus enhancing anti-CTLA-4 and anti-PD-1 therapeutic effects (26). However, in clinical practice, patients with metastatic colorectal cancer did not respond well; therefore, CAF-targeting drugs based on FAP^+^ failed the phase II trials (27, 28). Currently, relevant studies on this CAF inhibition strategy in GC treatment are lacking. Hence, exploring the matrix factors related to CAF in GC has clinical significance.

Weighted gene co-expression network analysis (WGCNA) is a powerful method for identifying co-expressed gene modules that are associated with specific biological traits or conditions (29). Once the network is constructed, modules or clusters of co-expressed genes are identified using hierarchical clustering or other methods. The modules are then analyzed for their relationship with different biological traits or conditions using correlation analysis, regression analysis, or other statistical methods (30). By analyzing gene expression data from multiple samples, WGCNA can provide insights into the underlying biological processes involved in disease pathogenesis or treatment response, and can identify new candidate genes or pathways for further investigation (31). Currently, the significance of WGCNA lies in its ability to identify co-expressed CAF-related gene modules that are associated with specific biological traits or conditions (32, 33); however, its use in GC for CAF and matrix infiltration remain unexplored. In this study, for the first time, we examined the hub modules most associated with matrix CAF penetration and used the Least Absolute Shrinkage and Selection Operator (LASSO) Cox regression analysis for further investigations. The results revealed that the CAF model could be a promising new therapy in GC.

## Methods

### Data acquisition and processing

The RNA-seq data and corresponding prognosis data of 330 TCGA gastric adenocarcinomas (TCGA-STAD) were downloaded from TCGA. In addition, normalized expression data and clinicopathological profiles for all GC samples in the GSE15459, GSE34942, GSE38749 and GSE84437 datasets from the GEO database have been integrated and included.

### Calculation of riskscore and immune infiltration related to the CAF Model

The estimated immune and cancer cell proportion (EPIC) algorithm, which is based on cell type deconvolution, the xCell algorithm, which is based on gene feature enrichment and the microenvironmental cell population counter (MCP-counter), which is based on the gene expression and tumor immune dysfunction and rejection (TIDE) algorithm, were used for CAF infiltration analyses (34). Using expression data, the ESTIMATE algorithm was used to estimate stromal cells and immune cells in malignant tumor tissue and calculate the stromal score, which indicates the level of stromal infiltration in each sample (35). Moreover, CAF infiltration, co-expression network and hub genes for matrix score were determined and identified using WGCNA (36, 37). Pearson correlation was used to compute the similarity matrix between any pair of genes. Then, the adjacency matrix was used for clustering and assessing the Pearson correlation between CAF infiltration, quantified by MEs and EPIC, and the matrix score, and selecting the most relevant modules for further analysis.

### Chemotherapy and immunotherapy response prediction

Using the Genomics of Cancer Drug Sensitivity (GDSC) (https://www.cancerrxgene.org/) database, the half-maximal inhibitory concentration (IC50) values of common drugs were estimated from the transcriptome data of each GC sample. TIDE (Tumor Immune Dysfunction and Exclusion) is a computational algorithm developed by researchers at the Dana-Farber Cancer Institute to predict the response of cancer patients to immune checkpoint inhibitors (ICIs) (38). ICIs are a class of cancer immunotherapy drugs that can help the immune system recognize and attack cancer cells. However, not all patients respond to ICIs, and TIDE was developed to identify patients who are unlikely to benefit from this treatment. TIDE works by analyzing gene expression data from tumor samples and predicting the likelihood of two types of immune dysfunction: T cell dysfunction and T cell exclusion. Furthermore, differences in TIDE score between CAF high or low-risk clusters were assessed using unpaired t tests, and the predictive power of CAF risk signatures was assessed using receiver operating characteristic curves and area under the curve (AUC) values.

### Statistical analysis

All statistical analyses were performed using R software and SPSS software. The median CAF risk score was the cutoff for each cohort to classify patients with GC (36, 37). Finally, the wilcoxon test was used for pairwise comparisons. P value less than 0.05 was considered statistically significant.

### Immunohistochemistry staining analysis

IHC was implemented with an anti-SPARC antibody-N-terminal at a 1:500 dilution per the manufacturer’s instructions (39). Based on the IHC staining intensity and density, digital image analysis is a powerful tool for quantifying IHC staining. It involves the acquisition of high-resolution digital images of stained tissues and the analysis of these images using specialized software. Several parameters were measured, including staining intensity, percentage of positive cells, and spatial distribution of staining.

### Implementing cox regression and nomogram establishment

Relevant data, including patient demographics, clinical characteristics, treatment details, and survival outcomes, were collected from TCGA and GEO databases. The data was cleaned, organized, and prepared for analysis, involving removing missing data, creating new variables, and categorizing continuous variables. Cox regression is a statistical method used to analyze survival data and identify factors that are associated with survival outcomes. In R language, the “coxph” function in the “survival” package was implemented for Cox regression analysis. This function requires specifying the outcome variable (time to event), the predictor variables, and any relevant covariates. A nomogram is a graphical representation of a statistical model that can be used to predict the probability of an event. In R language, the “rms” package was used to develop a nomogram based on the Cox regression model. This package requires specifying the predictor variables and any relevant covariates, as well as the time-to-event variable and censoring variable.

## Results

### Higher CAF infiltration suggests poorer overall survival in patients with GC

**Figure 1:**
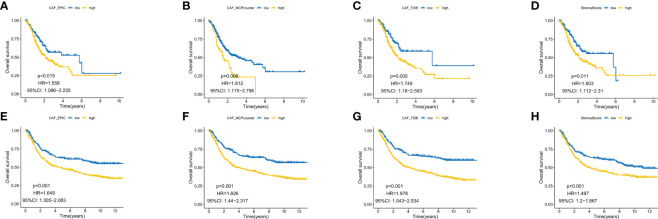
Kaplan–Meier analysis revealed that higher CAF infiltration was associated with poorer overall survival in patients with gastric cancer (GC) in both the GEO **(A–D)** and TCGA database **(E–H)**.

EPIC, MCP counter and TIDE algorithms were used to multiply and predict CAF infiltration, and the matrix score was calculated to predict the prognostic value on overall survival (OS). The expression level of CAF in the TCGA-STAD cohort and GEO datasets was determined and Kaplan–Meier (KM) curves were drawn (**Figures 1A–H**). A higher level of CAF infiltration was observed in both cohorts and significantly correlated with poor OS in patients with GC, highlighting the therapeutic potential of CAF in the prognosis of patients with GC. Therefore, it is necessary to further explore the correlation between CAF and GC matrix-related genes.

### Co-expression model of CAF and matrix scores and functional analysis of hub genes

**Figure 2:**
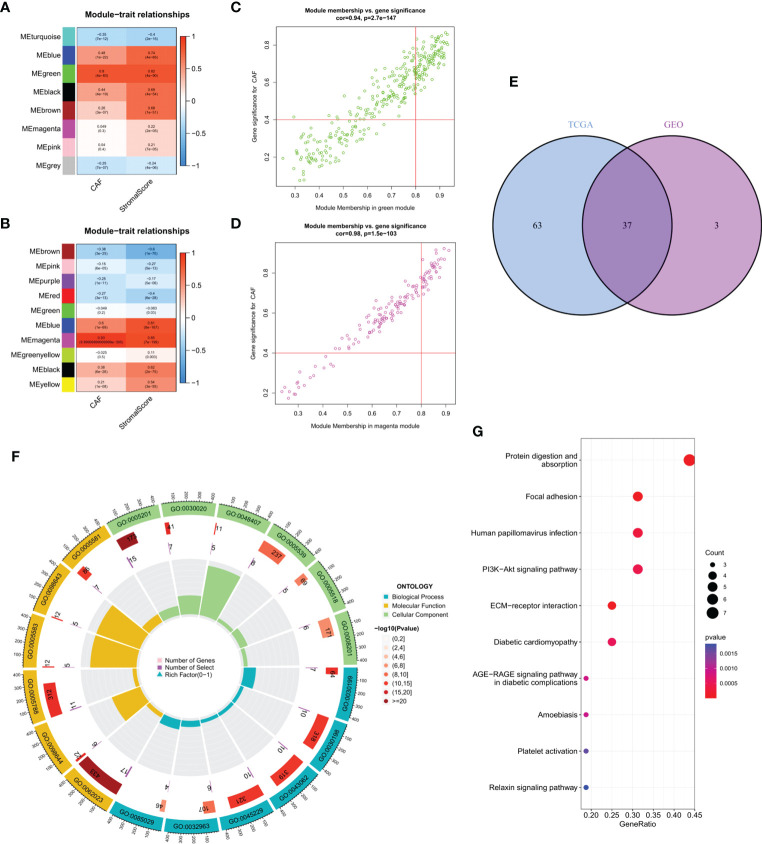
**(A)** The correlation between gene module eigengene and phenotype in the GEO database. **(B)** The correlation between gene module eigengene and phenotype in TCGA database. **(C)** Scatter plots of the MM and GS of each gene in the green module in GEO database. **(D)** Scatter plots of the MM and GS of each gene in the magenta module in TCGA database. **(E)** The intersection of GEO green and TCGA magenta module genes is presented in the Venn diagram. **(F)** Functional enrichment analysis in GEO, with blue, yellow and green representing BP, MF and CC terms, respectively. The color fill in the grey area represents the degree of enrichment. **(G)** Bubble diagram of functional enrichment analysis in TCGA.

WGCNA of the two database samples revealed a total of eight modules that were clustered in the GEO database, with the green module having the strongest relationship with CAF ratio (Cor = 0.8, p = 4e-83) and matrix score (Cor = 0.82, p = 4e-90) (**Figure 2A**). For TCGA, a total of 10 modules were co-polymerized, with the magenta module exhibiting the strongest relationship with CAF ratio (Cor = 0.93, p < 0.001) and matrix score (Cor = 0.85, p = 7e-199) (**Figure 2B**). Subsequent analyses of the green module (**Figure 2C**) and magenta module (**Figure 2D**) were performed. The scatter plots showed a strong correlation between module membership and gene significance for CAF and interstitial score.

Venn diagram of the intersection of the two hub gene sets identified a total of 37 common genes, which were then analyzed using GEO and KEGG (**Figure 2E**). Extracellular matrix organization, collagen-containing extracellular matrix and extracellular matrix structural constituent were the main enriched BP, CC and MF terms, respectively (**Figure 2F**). Protein digestion and absorption, focal adhesion and human papillomavirus infection were the major pathways enriched in KEGG (**Figure 2G**).

### Construction of a prognostic risk model based on the matrix CAF

**Figure 3:**
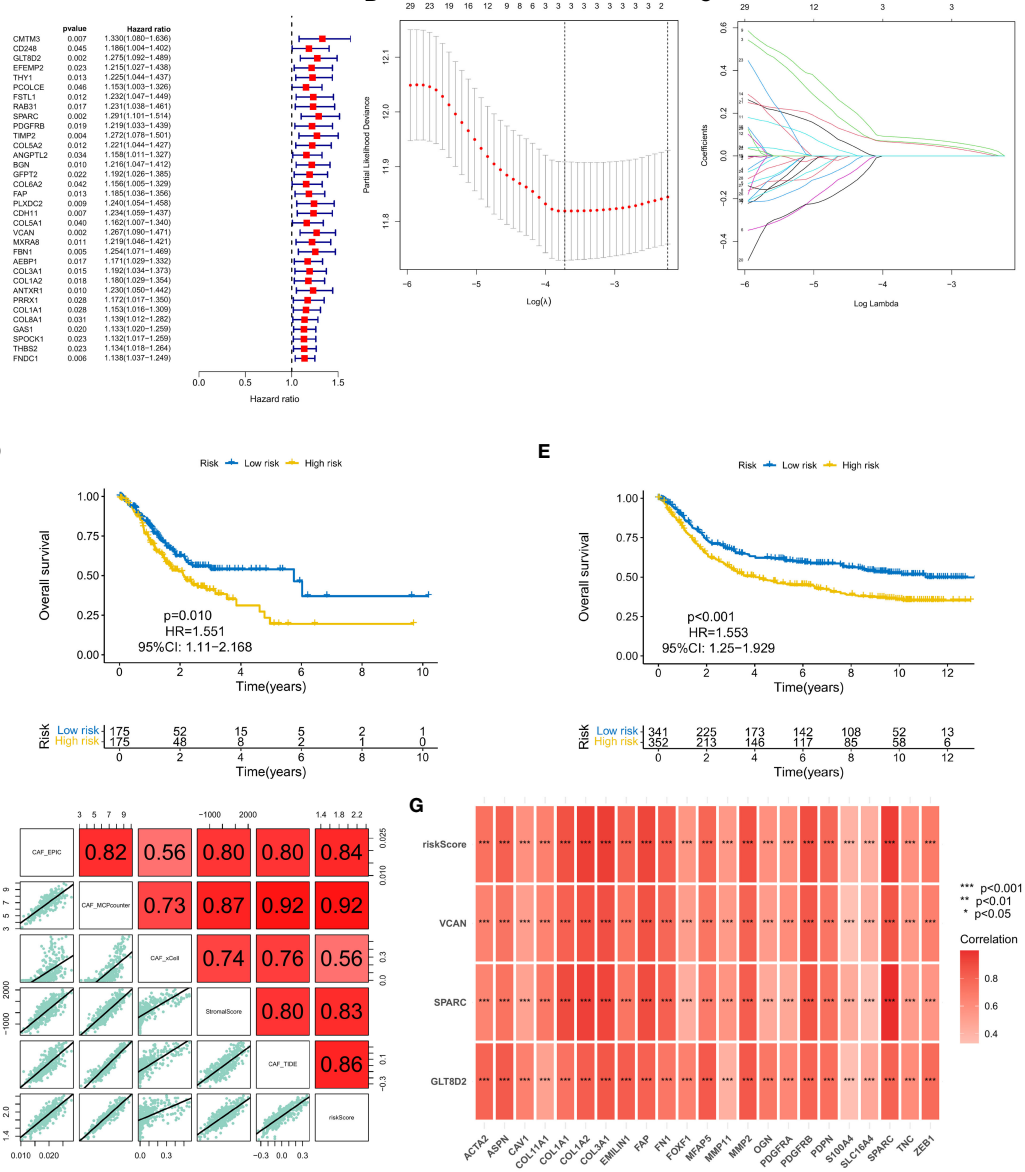
**(A)** Univariate Cox regression analysis. **(B, C)** LASSO regression analysis. **(D, E)** Survival analysis of the high CAF risk group and low CAF risk group in GEO and TCGA, respectively. **(F, G)** Spearman correlation analysis on predictions of CAF risk score, matrix score, EPIC, MCP counter, xCell and TIDE for CAF abundance.

**Figure 4:**
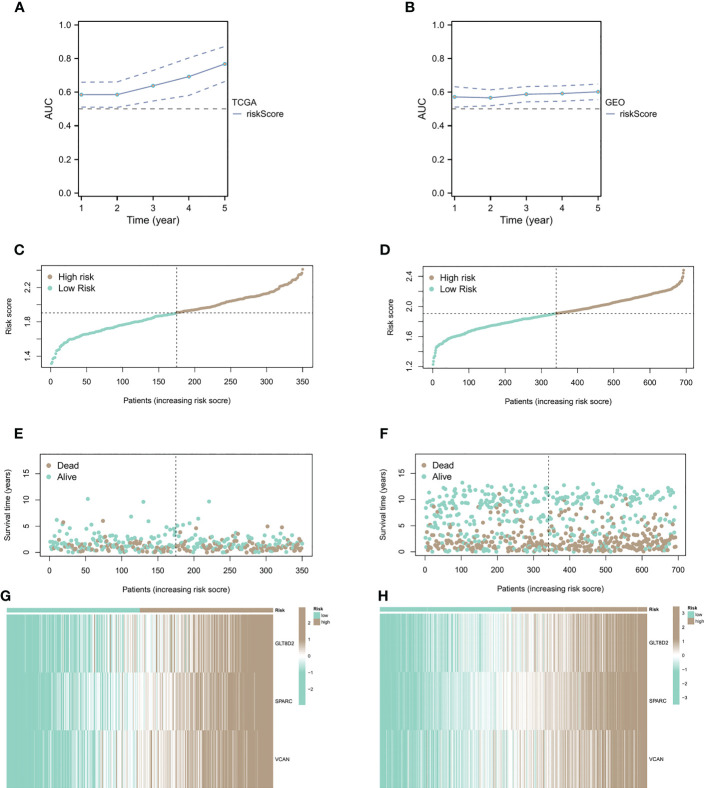
Relationship between CAF risk assessment and prognosis. **(A, B)** CAF risk assessment analysis on patients from sample sources in TCGA and GEO databases. **(C-F)** Patients with high CAF risk had higher risk scores and shorter survival times than the low CAF risk group. **(G, H)** The correlation heatmap of the distribution of key genes.

A total of 37 common hub genes were screened using univariate Cox regression analysis, and 34 OS-related genes with p < 0.05 were analyzed using LASSO regression (**Figures 3A-C**). Finally, three genes were identified to construct the CAF risk model: CAF risk score = GLT8D2 expression * 0.066 + SPARC expression * 0.087 + VCAN expression * 0.061. Using this risk model, patients with GC in each cohort were divided into high and low CAF risk groups, with a bit risk score selected as the threshold. The KM curve showed that for patients with GC, the OS of the high CAF risk group was worse than that of the low CAF risk group in GEO (p = 0.010, hazard ratio (HR) = 1.551, 95% confidence interval (CI): 1.11–2.168) (**Figure 3D**) and TCGA (p < 0.001, HR = 1.553, 95% CI: 1.25–1.929) databases (**Figure 3E**). These results suggest that CAF and stroma-related signature genes are key prognostic markers for GC. To further verify the robustness of the CAF model as a predictor of CAF permeability, Spearman correlation evaluated CAF abundance prediction using CAF risk score, matrix score, EPIC, MCP counter, xCell and TIDE (**Figures 3F, G**). The results consistently showed that CAF risk scores in GEO (**Figure 4F**) and TCGA (**Figure 4G**) databases were significantly positively correlated with various CAF permeability scores.

### Relationship between CAF risk assessment and prognosis

We conducted CAF risk assessment analysis on patients from sample sources in TCGA and GEO databases (**Figures 4A, B**), observing that the prolongation of patients’ survival time was associated with higher risk score AUCs, indicating that the CAF model is effective in predicting patient survival in the long run. Additionally, patients with high CAF risk had higher risk scores (**Figures 4C, D**) and shorter survival times than the low CAF risk group (**Figures 4E, F**). The correlation heatmap shows the distribution of key genes in the high and low groups (**Figures 4G, H**).

### Association of CAF risk score with immune cell aggregation

**Figure 5:**
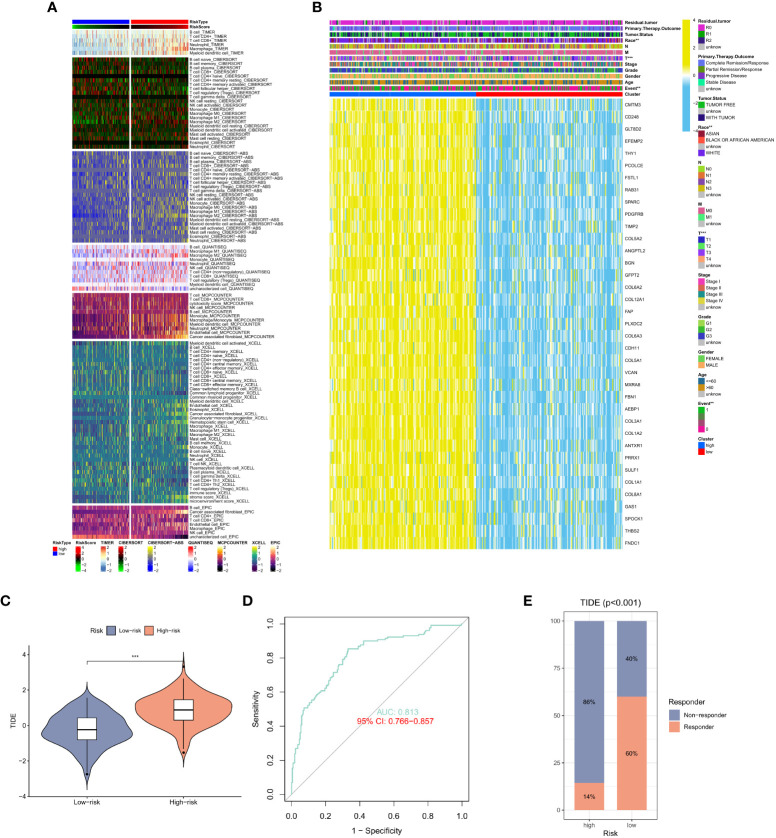
**(A)** Heat map of CAF risk score in relation to immune cells. **(B)** Heat map of the relationship between CAF risk score and race, T stage, event and other factors. **(C)** Differences in TIDE scores in the high and low CAF risk groups. **(D)** ROC curve analysis. **(E)** The proportion of immune response and TIDE score in the high and low CAF risk groups.

After studying the relationship between CAF expression and prognosis, we shifted our focus to immune cells. Similar results were observed in GC sample analysis using different algorithms. Using CIBERSORT and TIMER algorithms, in immune cells in the high CAF risk group, we found that the activity of T cell CD4+ memory activated and macrophage, particularly CD8+ T cell, CD4+ T cell, were significantly lower than that of the low CAF risk group (**Figure 5A**). Meanwhile, we also analyzed the relationship between race, T stage, event and CAF risk score. The high CAF risk group was significantly associated with multiple factors (**Figure 5B**) and a high TIDE score (**Figure 5C**). Moreover, the ROC curve showed a reliable score (AUC = 0.813) (**Figure 5D**) for the high CAF risk group also and a higher proportion of non-response to immunotherapy (**Figure 5E**). These findings suggest that patients with GC having high CAF risk could exhibit a worse immunotherapy response.

### GSEA of the CAF signature

**Figure 6:**
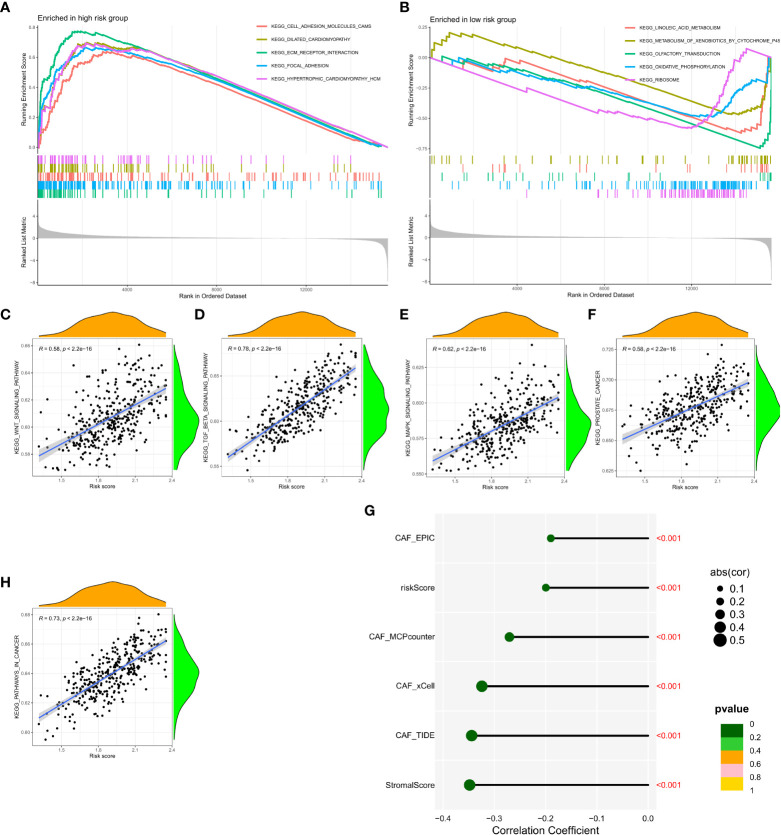
**(A, B)** Gene set enrichment analysis (GSEA) of KEGG gene sets between the high and low CAF risk groups. **(C-F, H)** Spearman correlation analysis between enrichment of functional pathways and risk scores. **(G)** Spearman correlation analysis revealed the negative correlation between TMB and CAF activator.

Gene Set Enrichment Analysis (GSEA) was performed in the GEO and TCGA datasets between high and low CAF risk groups to elucidate the functional enrichment of CAF characteristics. The main KEGG enrichment signaling pathway of the high-risk group genes was observed to be cell adhesion molecules, dilated cardiomyopathy, ECM receptor interaction, focal adhesion and hypertrophic cardiomyopathy (**Figure 6A**). Additionally, the KEGG enrichment signaling pathways mainly included linoleic acid metabolism, metabolism of xenobiotics by cytochrome P45, olfactory transduction, oxidative phosphorylation and ribosome (**Figure 6B**). These pathways were further examined to determine their association with CAF risk score, revealing a positive correlation between risk score and the rich integration of KEGG pathways, including the WNT signaling pathway, β signaling pathway, MAPK signaling pathway, prostate pathway and pathways in cancer (**Figures 6C-F, H**). Spearman correlation analysis revealed that the tumor mutation burden (TMB) was negatively associated with MCPcounter, xCell, TIDE, and other activators (**Figure 6G**).

### CAF and drug sensitivity analysis

**Figure 7:**
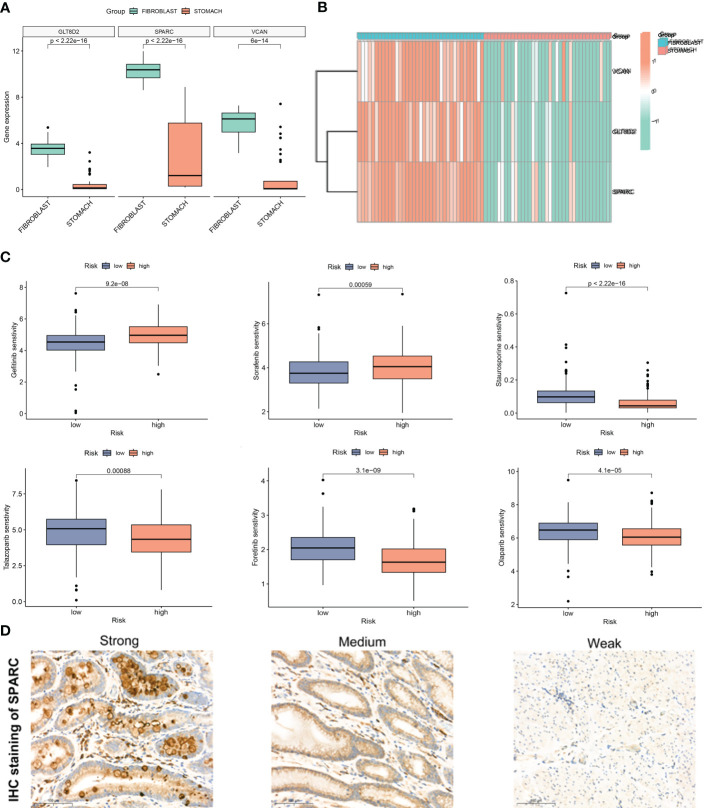
**(A, B)** Expression difference of *GLT8D2, SPARC* and *VCAN* in gastric cancer (GC) fibroblasts and normal cells. **(C)** Susceptibility to different drugs in high and low CAF risk groups. **(D)** IHC staining of *SPARC* in GC tissue.

To validate the efficacy of selected key signatures in treatment, we performed the expression differences of the three hub genes *GLT8D2, SPARC* and *VCAN* in GC fibroblasts and normal cells. The expression levels of these genes were markedly high in the fibroblasts (**Figures 7A, B**). We then conducted sensitivity tests for different drugs in the CAF risk groups, with IC50 as the evaluation criteria. Except for Gefitinib and Sorafenib, other drugs showed higher sensitivity in the low CAF risk group, indicating an effective treatment response (**Figure 7C**). Moreover, IHC revealed high staining levels of SPARC (Weak, Medium, and Strong staining) in GC tissues (**Figure 7D**).

### Prediction of clinical features using the CAF model

**Figure 8:**
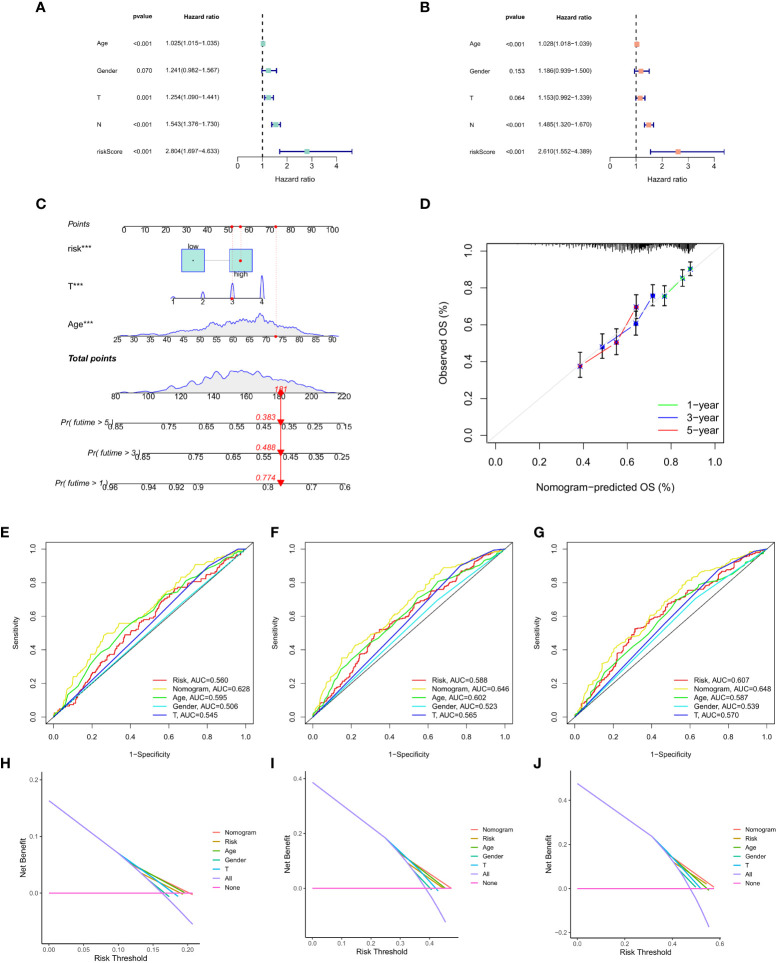
**(A)** Univariate Cox regression analysis. **(B)** Multivariate Cox regression analysis. **(C)** Nomogram of risk score, T stage and age. **(D)** 1-, 3- and 5-year survival prediction of patients with GC based on the nomogram. **(E-G)** ROC curve analysis based on all factors. **(H-J)** DCA decision curve.

Finally, univariate Cox regression analysis was used to determine whether CAF was significantly correlated with T stage, N stage, age, risk score and gender (**Figure 8A**). Multivariate regression analysis was applied to verify if risk score had the strongest association with CAF (p < 0.01, HR = 2.804; **Figure 8B**). Then, we focused on risk score, T stage, age and total score to construct a nomogram for prognosis prediction (**Figure 8C**). Meanwhile, 1-, 3- and 5-year survival prediction curves were drawn using the most significantly related factors, i.e., risk score, T stage and age. as Additionally, ROC curve analysis was performed for all factors (**Figures 8D-G**). The results showed that short-term survival prediction was more effective for the predictive CAF model. Finally, the DCA decision curve revealed that the model has a good predictive effect (**Figures 8H-J**).

## Discussion

Extensive fibrosis is common in GC, especially in undifferentiated GC. Cancer-associated fibroblasts (CAFs) are a major component of the tumor microenvironment and play a critical role in the development, progression, and drug resistance of gastric cancer. Massive infiltration of CAFs leads to TME resistance to tumor lymphocyte invasion, thereby promoting GC progression, immunosuppression and therapeutic resistance (40, 41). Various studies report CAFs are associated with poor prognosis and aggressive tumor behavior in gastric cancer. Several studies have shown that the expression of CAF-related markers such as α-smooth muscle actin (α-SMA), fibroblast activation protein (FAP), and platelet-derived growth factor receptor-α (PDGFR-α) can be used as diagnostic and prognostic markers for gastric cancer. Thus, improving stromal CAF-targeting therapies by developing new molecular targets in cancers is vital (42, 43).

In this study, we explored mutual CAF and matrix co-expression networks in multiple GC queues using WGCNA and multiple calculations. A three-gene (*GLT8D2, SPARC* and *VCAN*) prognostic CAF model was developed and verified by machine-learning algorithms. Moreover, markedly elevated TMB levels were found in patients from CAF^low^ cluster suggesting that the CAF model may serve as a biomarker for the immunotherapy stratification of GC. Immunotherapy has shown promising findings in the treatment of gastric cancer. However, the response rate to immunotherapy is limited, and CAFs have been shown to play a role in this resistance. CAFs can create a physical barrier that prevents T cells from reaching cancer cells and can also secrete immunosuppressive cytokines and growth factors that inhibit T cell function. Targeting CAFs has been proposed as a strategy to overcome this resistance and enhance the effectiveness of immunotherapy (44).

CAFs contribute to drug resistance in gastric cancer through various mechanisms, including the secretion of cytokines and growth factors that promote tumor growth and survival, the activation of signaling pathways that promote drug resistance, and the deposition of extracellular matrix proteins that create a physical barrier to drug penetration. Targeting CAFs can overcome drug resistance in gastric cancer and improve patient outcomes (44). Importantly, this study is the first to reveal a negative association between TMB levels and infiltration in patients with CAF activators and GC. Therefore, we hypothesize that higher TMB could produce a strong tumor-killing effect by regulating the weak local microenvironment of stromal fibroblasts. However, further studies are needed to clarify the interaction between TMB and CAF infiltration (43).

In addition, our findings revealed that cell adhesion molecules, ECM receptors and focal adhesion pathways were largely enriched in the CAF^high^ cluster, and single-sample GSEA also revealed that CAF risk scores were positively correlated with WNT, TGF-β and MAPK enrichment scores. Moreover, TGF-β signaling has been demonstrated to play a role in CAF activation (45–47), and similarly, CAFs can regulate and maintain the stemness of GC cells through TGF-β signalling (48). Pathological angiogenesis is widely described as a key process that can expand cancer tissue and GC cell invasion and metastasis (49–51). To ensure the robustness of the model, we quantified CAF infiltration in GC using four bioinformatics methods: EPIC, xCell, MCP counter and TIDE. The expression of the three genes was significantly increased in fibroblasts, demonstrating that these genes can act as specific markers of CAF in GC. Therefore, the CAF model was significantly associated with the infiltration of CAFs, so that the CAF labelling could accurately assess CAF infiltration level in samples with GC.

Regarding the three hub genes, *GLBT2* plays an important role in pulmonary arterial hypertension (PAH) and participates in immune processes and ECM functions as a hub gene, highlighting its therapeutic potential in PAH (52). Additionally, in the liver, *GLT8D2* can positively regulate the expression of the ApoB100 protein in hepatocytes, promote the stable secretion of very low density lipoprotein and reduce the accumulation of triglycerides in hepatocytes, thus reducing the incidence of non-alcoholic fatty liver disease (NAFLD) (53). *SPARC* has been previously reported as a potential target in GC (54, 55). The high expression of *VCAN* can be used as an independent predictor to indicate the adverse outcome of GC (56). It also exhibits a significantly high expression in hepatocellular carcinoma induced by hepatitis B, which is significantly correlated with poor prognosis and immune suppression and can be used as a potential biomarker for hepatocellular carcinoma. Nevertheless, the functions of these genes in GC CAFs remains poorly explored. Therefore, future explorations *in vivo* and *in vitro* are essential for understanding the potential role of GC-related CAFs in invasiveness, drug resistance and immunosuppression.

Certain limitations exist in this study. First, we did not cross-verify the true clinical value of the established CAF model. Second, no animal experiments were conducted to verify the key role of CAF-specific signatures in the progression of gastric cancer. Finally, our model construction can only be considered as a guide for future CAF studies in GC. CAFs act as an essential role in the TME of gastric cancer, and have been shown to interact with immune cells, including T cells, natural killer (NK) cells, and dendritic cells (9). CAFs can modulate the immune response through the secretion of cytokines and growth factors and can also affect the recruitment and function of immune cells. Targeting CAFs can alter the tumor microenvironment and enhance the immune response to cancer cells (10). Several therapeutic strategies targeting CAFs have been developed for the treatment of gastric cancer, including the use of small molecule inhibitors, monoclonal antibodies, and RNA interference-based approaches (57). For example, targeting CAFs with the anti-fibrotic agent pirfenidone has been shown to increase the sensitivity of gastric cancer cells to chemotherapy and immunotherapy.

## Conclusion

In this study, the high infiltration of CAF in TME was associated with a worse GC prognosis. Furthermore, three hub genes, *GLT8D2, SPARC* and *VCAN*, were identified as prognostic biomarkers of CAF, which were then used to establish a CAF model based on these three genes. The infiltration, prognosis, immunotherapy resistance and drug resistance of CAF in patients with GC were evaluated, which provided a new framework for further exploration of CAF in GC. Targeting CAFs represents a promising therapeutic strategy for enhancing the effectiveness of immunotherapy and improving outcomes for patient with GC.

## Data availability statement

The original contributions presented in the study are included in the article/supplementary material, further inquiries can be directed to the corresponding author/s.

## Ethics statement

The studies involving human participants were reviewed and approved by ethics committee of Fudan University Shanghai Cancer Center. The patients/participants provided their written informed consent to participate in this study.

## Author contributions

Conceptualization, data curation and formal analysis: LG, DD, and CW. Funding acquisition: LG. Investigation and methodology: LG, DD, and CW. Resources and supervision: DZ. Validation and visualization: LG, DD, and CW. Original draft: LG and DD. Editing: CW and DZ. All authors contributed to the article and approved the submitted version.
